# New Jersey and Vicinity

**Published:** 1897-07

**Authors:** E. Buckley

**Affiliations:** Secretary; East Orange, N. J.


### THE VETERINARY MEDICAL SOCIETY OF THE STATE OF NEW JERSEY AND VICINITY.

THE regular monthly meeting was held at Dr. L. R. Sattler's hospital in Newark on Tuesday, June 8th. Called to order at 4 P.M., President Sattler in the chair. At the roll-call the following members responded: Drs. Sattler, Ogden, Ancker, Smith, Knott, and Buckley. The minutes of the previous meeting were read and approved. Reports of Secretary and Treasurer were read and adopted. It was moved and seconded that the next meeting be held at Dr. Ogden's residence in Orange, N. J.

Discussions and reports from practice were next in order. Dr. Ancker reported a case of tetanus with complete trismus; 12 grains of Gibier's antitetanine were given in six doses in forty-eight hours, with 50 grains of chloral hydrate in solution per rectum. The animal made a complete recovery.

In discussing laminitis, Dr. Ancker gave his treatment as follows: 8 decigrammes of pilocarpin in three doses in twenty-four hours, also salicylate soda, 60 grains in twenty-four hours. In three days the animal is generally able to move about. In discussing the treatment of worms in the horse Dr. Sattler recommended the following:

| | |
|---|---|
| ℞.—Pulv. arsenici alb. | 1 |
| Tart. stil. | 20 |
| Pulv. aloes | 30 |
| Pulv. althææ | 30 |

M. Fiat pil. no. ij. S.—Give the two pills twelve hours apart.

Dr. Sattler promised to explain an interesting case of rheumatic endocarditis at the next meeting.

E. BUCKLEY,
Secretary.

EAST ORANGE, N. J.

